# Is bariatric surgery a solution for obesity?

**DOI:** 10.1186/1753-6561-6-S3-P22

**Published:** 2012-06-01

**Authors:** Mary Hogan

**Affiliations:** 1 University of Texas, San Antonio, Texas 78249, USA; 2 Texas Center for Medical & Surgical Weight Loss, San Antonio, Texas 78217, USA

## Background

The prevalence of obesity in the United States continues to grow at an exponential rate. Obesity is a risk factor for a variety of chronic conditions including diabetes, hypertension, high cholesterol, stroke, heart disease, certain cancers, and arthritis [2]. The CDC released a report in July 2009 stating the cost of health care related to obesity is as high as $ 147 billion annually. In 1998 6.5 percent of medical costs were a result of obesity; in 2009, 9.1 per cent of annual medical costs were a result of obesity-related diagnosis; in 1998 this was 6.5 per cent. The medical costs for an obese person are 42 per cent higher than for a person of normal weight or approximately $1,429 per year [1].

## Materials and methods

The purpose of this research is to determine the possible medical benefits of bariatric surgery for patients with a BMI of 35 or greater. The dataset includes 1,217 patients of whom 42 percent are White, 40 percent are Hispanic, 7.2 percent Black, 5.6 percent Pacific Islander, and 2.5 percent Other. The range of age of the patients is 18-75 years. Four different surgical procedures were performed and patients; and the post-operative period ranges from two to 51 months. A substantial portion of patients were morbidly obese (85 percent) prior to surgery, only 15 percent had a BMI below 40. The data gathered included whether the patient was diagnosed and being treated for a specific co-morbidity. The range included cardiac problems, high cholesterol, hypertension, diabetes, incontinence, joint problems, psychological disorders, and respiratory problems. This study focused on those co-morbidities affecting the cardiovascular system, cholesterol, high blood pressure, diabetes and respiratory. The first analysis was completed for individuals that presented with high-cholesterol. The second analysis was completed for individuals that presented with diabetes. The third analysis was for individuals presenting with hypertension. The last analysis was for individuals presenting with respiratory disease co-morbidities. The predictor variables of interest are initial BMI, BMI at 12 months, and type of procedure.

## Results

The results of this study show that bariatric surgery reduces BMI; levels of obesity, and reduces the incidence of co-morbidities. The bypass surgery was most effective with patients achieving weight loss to a normal level. Patients found improvement and/or resolution of pre-existing cardiac issues, high cholesterol, high blood pressure, Type 2 diabetes, and sleep apnea.

## Conclusions

Medical costs attributable to obesity are almost entirely a result of costs generated from treating the diseases that obesity promotes, and as long as obesity prevails to the extent that it does today, it will continue to be a significant burden on health care. This research has found improvement in the presence of co-morbidities in post-operative bariatric surgery patients.
